# Evolution Mechanisms of Flow and Transient Temperature Fields in Wet Friction Pair with Bionic Hexagonal Micro-Texture

**DOI:** 10.3390/biomimetics11040271

**Published:** 2026-04-15

**Authors:** Donghui Chen, Yulin Xiao, Shiqi Hao, Chong Ning, Xiaotong Ma, Bingyang Wang, Xiao Yang

**Affiliations:** 1 Key Laboratory of Bionic Engineering, Ministry of Education, Jilin University, Changchun 130022, China; dhchen@jlu.edu.cn (D.C.); ylxiao22@mails.jlu.edu.cn (Y.X.); haosq23@mails.jlu.edu.cn (S.H.); ningchong23@mails.jlu.edu.cn (C.N.); xtma23@mails.jlu.edu.cn (X.M.); bywang2024@nbut.edu.cn (B.W.); 2 College of Biological and Agricultural Engineering, Jilin University, Changchun 130022, China; 3 Weihai Institute for Bionics, Jilin University, Weihai 264401, China; 4 College of Mechanical and Automotive Engineering, Ningbo University of Technology, No. 769, Binhai Second Road, Hangzhou Bay New District, Ningbo 315336, China

**Keywords:** bionic micro-texture, wet friction pair, flow dynamics, thermo-hydrodynamic performance

## Abstract

Friction pairs in wet clutches operate under complex conditions, which can cause surface damage and reduce overall clutch reliability. Surface texturing is an established technique for improving the tribological performance of such mechanical interfaces. Inspired by the wet adhesion properties of tree frog foot pads, a bionic regular hexagonal micro-texture was designed on the mating steel plate. A three-dimensional transient computational fluid dynamics (CFD) numerical methodology was developed and rigorously verified via pin-on-disc friction experiments. Subsequently, this verified numerical framework was extrapolated to establish disc-on-disc CFD models. The results demonstrated that the bionic hexagonal micro-texture altered flow field characteristics, increasing the local maximum flow velocity by 7.9% compared to untextured surfaces. Furthermore, the micro-textured grooves expanded the effective area for convective heat transfer and facilitated local fluid exchange, reducing the maximum average bulk temperature by 20.5% and the maximum radial temperature by 20.7%. Adjusting the structural parameters of these micro-textures further regulated the interfacial flow and temperature fields; notably, deeper grooves induced vortices at land region edges, accelerating flow velocity and decreasing the overall radial temperature gradient. This study provides a theoretical reference for enhancing the thermo-hydrodynamic performance of wet clutch friction pairs.

## 1. Introduction

Wet clutches serve as essential power transmission components in tractors [1]. As the core element within these systems, the wet friction pair is subjected to severe operational conditions, including high mechanical loads, elevated relative sliding velocities, and intense thermal shocks at the contact interfaces [2]. Consequently, the thermal and hydrodynamic performance of the wet friction pair directly determines the torque transmission capacity, service life, and overall reliability of the wet clutch [3]. If the generated frictional heat is not efficiently dissipated by the lubricating oil, it leads to severe localized temperature rise, which reduces the dynamic viscosity of the lubricant, weakens the oil film load-carrying capacity, and accelerates the thermal degradation of the friction material [4,5,6]. Processing the contact interfaces of the friction pair with surface texturing has emerged as a promising technical approach to enhance convective heat transfer and improve lubrication characteristics [7]. By fabricating specific geometric topographies on the contact interface, this technology can significantly modify the hydrodynamic pressure distribution and the development of the thermal boundary layer in the near-wall fluid region. This method has demonstrated distinct advantages across numerous industrial applications, such as reducing the friction and wear of cutting tools [8], improving the hydrodynamic performance of thrust bearings [9], mitigating the severe localized thermal accumulation on friction pairs [10], and enhancing the tribological behaviors of wet clutches [11,12].

To minimize thermal damage, introducing macroscopic grooves on the friction surfaces is an effective approach to facilitate lubrication and heat dissipation. Extensive numerical studies have systematically investigated the guiding effects of traditional macroscopic grooves, such as radial grooves [13], waffle grooves [13], and bidirectional grid grooves [14], on the fluid flow trajectories, hydrodynamic pressure distributions, and overall temperature rise within the wet clutch [15]. Z et al. [16] conducted a coupled thermal–fluid analysis, verifying that macroscopic grooves can reduce the average bulk temperature of the wet friction pair. By establishing a coupled thermal–fluid model for a grooved disk system, Wu et al. [17] elucidated the specific impacts of rotational speed, forced inlet flow rate, and oil temperature on the macroscopic temperature distribution. Based on finite element heat transfer models, Li et al. [18] and Bian et al. [19] further explored how different material properties and thermal boundary conditions affect the temperature field of friction pairs equipped with macroscopic grooves. Wang et al. [20] and Liao et al. [21] respectively fitted and modified the calculation formulas for the convective heat transfer coefficient under various macroscopic groove conditions by incorporating cavitation effects or utilizing the response surface methodology. Tan et al. [22] utilized a coupled thermal–fluid–solid simulation combined with optimization algorithms to analyze and refine the structural parameters of radial and annular composite oil grooves in high-speed helicopter clutches, which effectively reduced the drag torque and temperature rise. Furthermore, recent advancements have expanded the geometric design of macroscopic grooves beyond conventional radial or waffle topologies by coupling novel shapes with advanced multi-objective optimization algorithms. For instance, Rogkas et al. [23] evaluated innovative mid-relief and arc-bow groove topologies using a data-driven Gaussian Process Regression model and exhaustive search optimization. Their results demonstrated that optimized groove geometries can synergistically minimize viscous drag power loss while preserving torque capacity. Similarly, Wu et al. [24] employed an Atom Search Optimization–Random Forest model combined with the Non-dominated Sorting Genetic Algorithm II to optimize the cross-sectional profiles of grooves. This multi-objective optimization approach effectively achieved a simultaneous reduction in drag torque and a significant enhancement of the localized convective heat transfer coefficient. While these studies extensively discussed the positive contributions of macroscopic grooves to flow field evolution and heat transfer mechanisms, the fabrication of large-scale grooves inherently reduced the actual contact area of the friction pair. This reduction can compromise torque transmission stability and induce non-uniform local contact pressures. Consequently, surface micro-texturing technology has been increasingly adopted to ensure stable torque transmission while preserving lubricant performance and convective heat transfer capacity [25].

Surface micro-texturing technology plays an important role in improving the tribological performance of mechanical interfaces. Numerous studies have shown that Laser Surface Texturing (LST) technology can significantly optimize the comprehensive performance of various mechanical components. For instance, Ausas et al. [26] indicated that strategically designed micro-textures on sliding bearings effectively enhanced the load-carrying capacity of the fluid film and overall operational stability. In the context of internal combustion engines, Mezghani et al. [27,28,29] confirmed that applying LST to piston rings and cylinder liners mitigates friction and wear through the lubricant retention capabilities of the micro-textures. Building upon these prior findings, Wos et al. [30] numerically validated the benefits of surface micro-texturing in enhancing unidirectional hydrodynamic lubrication. Specifically regarding wet friction pairs, Nyman et al. [31] investigated circular, square, and triangular micro-textures on friction plates, demonstrating via CFD and experimental testing that these patterns generated micro-hydrodynamic effects, thereby increasing oil film load capacity and improving overall lubrication states. In summary, these fundamental studies validated the positive role of surface micro-texturing in enhancing hydrodynamic pressure and improving the lubrication performance at the contact interfaces of friction pairs.

To maximize the tribological performance of micro-textured surfaces, several studies have focused primarily on the optimization of micro-texture geometries. Tang et al. [32] optimized micro-textured surfaces using a level-set based approach, revealing that herringbone textures can enhance the load-carrying capacity and lubrication efficiency in parallel thrust bearings under cavitation conditions. Xu et al. [33] demonstrated that optimized micro-texture can increase the load-carrying capacity of SiC hybrid ceramic bearings by a factor of 2.5 compared to untextured surfaces. Experimental research by Kovalchenko et al. [34] showed that micro-dimple textures have a significant effect on improving tribological performance under severe conditions, such as high-speed, heavy-load, and high-viscosity lubricant. Furthermore, finite element analyses by Gherca et al. [35] emphasized that altering the geometric features of rotor textures substantially improved thrust bearing hydrodynamics. However, most micro-texture designs in these studies are confined to relatively simple, isolated geometries. Under the high-speed sliding conditions typical of wet clutch friction pairs, isolated micro-textures lack continuous fluid channels, which often leads to uneven temperature distributions across the contact surface.

Surface micro-textures exhibit a wide variety of forms; thus, determining an appropriate micro-texture pattern and investigating the influence of its structural parameters on tribological performance are important steps for enhancing the comprehensive performance of wet friction pairs. In recent years, bionics has offered a new perspective to address this challenge. To systematically exploit biological mechanisms for tribological enhancement, various bio-inspired surface textures have been comparatively assessed. For example, Rogkas et al. [36] conducted a comprehensive computational fluid dynamics analysis to evaluate engineered micro-textures inspired by biological prototypes, such as shark skin, lotus leaves, dung beetles, and dragonfly wings. Their comparative study revealed that pillar-based and grooved micro-geometries exhibit significant potential in manipulating fluid directionality and reducing viscous drag torque in rotating hydrodynamic lubrication regimes. However, the selection of a specific biological prototype must strictly align with the unique thermal and hydrodynamic demands of wet clutches. While isolated pillar or parallel ridge textures excel in unidirectional drag reduction under open-flow conditions, wet friction pairs require not only efficient drag management but also the rapid, omnidirectional expulsion of interfacial fluid to establish a stable mechanical contact area, alongside continuous fluid channels for intense convective heat dissipation. Consequently, the tree frog foot pad serves as a highly targeted and superior biomimetic prototype. In nature, amphibians such as tree frogs achieve exceptionally stable wet adhesion in moist environments because their epithelial surfaces have evolved into densely packed hexagonal columnar cells separated by an interconnected network of micro-channels [37]. This contiguous hexagonal topography acts as a highly efficient natural drainage system, swiftly expelling excess interfacial fluid to mitigate severe hydrodynamic repulsion, while the expansive hexagonal land regions ensure a maximized and uniform solid-to-solid contact area for stable torque transmission [38]. Inspired by this mechanism, Chen et al. [39] systematically investigated the effect of the hexagonal groove structural parameters on the wet friction characteristics of bionic structures on glass. By comparing them with square, rhombic, and triangular patterns, they demonstrated the higher frictional advantages of the bionic hexagonal structure. Iturri et al. [40] fabricated bionic hexagonal specimens of PDMS material with different aspect ratios. Compared to untextured specimens, the tribological performance of the bionic hexagonal specimens (specifically in terms of generating enhanced friction and maintaining stable interfacial adhesion) was noticeably improved on both wetting and non-wetting surfaces. Liu et al. [41] also reported enhanced oil film load-carrying capacity after applying hexagonal micro-textures to the stator–rotor friction pair of a screw pump.

While prior research validates the advantages of hexagonal micro-textures in enhancing the lubricant’s retention capacity, increasing the oil film’s load-carrying capacity and convective heat transfer, texturing the separator plate remains rarely reported. Consequently, a systematic quantitative analysis and mechanistic understanding of how micro-texture structural parameters (e.g., groove width, groove depth, and circumcircle diameter) reshape the three-dimensional micro-flow dynamics, and subsequently affect the radial temperature gradient within the transient temperature field, are still lacking.

Inspired by the highly efficient interfacial fluid drainage capabilities of tree frog foot pads, this paper investigated a wet friction pair consisting of a friction plate and a mating steel plate featuring bionic regular hexagonal micro-textures. Initially, a transient pin-on-disc computational fluid dynamics (CFD) model was established using ANSYS Fluent, and its underlying numerical methodology was meticulously verified via UMT TriboLab pin-on-disc experiments. Employing this experimentally verified numerical framework, a full-scale disc-on-disc CFD model was subsequently constructed. Based on the disc-on-disc simulations, the fluid dynamic characteristics and transient temperature field evolutions were compared between untextured and bionic textured friction pairs under the fully lubricated state. Furthermore, this study elucidated how the structural parameters of the bionic hexagonal micro-texture (i.e., groove width, groove depth, and circumcircle diameter) regulated the average flow velocity, hydrodynamic pressure, average bulk temperature, and transient radial temperature gradient. This study aims to provide a theoretical reference for the thermal and hydrodynamic performance design of wet clutch friction pairs.

## 2. Materials and Methods

**Figure 1:**
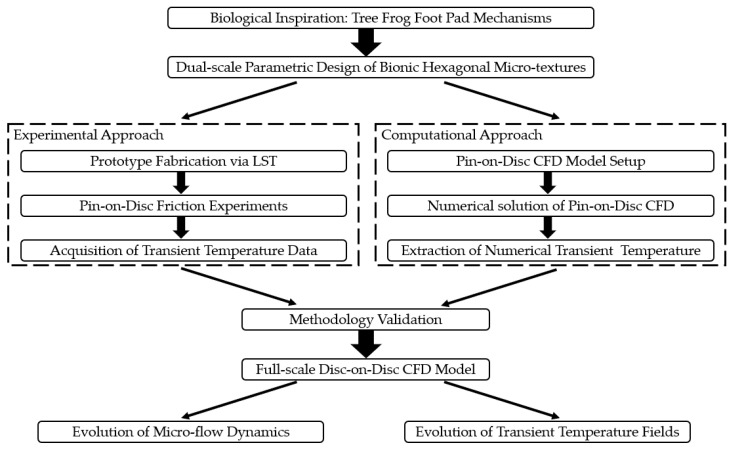
Overview of the workflow diagram in this study.

To systematically investigate the micro-hydrodynamic characteristics and transient thermal evolution mechanisms within a wet clutch friction pair featuring a bio-inspired hexagonal micro-textured mating steel plate, a progressive and multi-scale research framework was established, integrating both experimental and computational methodologies in Ansys 2025 R1. Initially, pin-on-disc friction experiments were conducted in parallel with the development of a pin-on-disc CFD model. The primary objective of the pin-on-disc experiments was strictly to validate the accuracy and reliability of the numerical algorithms, mesh generation strategies, and boundary conditions adopted in the CFD modeling. Upon successful experimental validation, this verified numerical methodology was extrapolated to construct a full-scale disc-on-disc CFD model. Ultimately, this validated disc-on-disc CFD model was utilized to analyze the regulatory effects of the bionic micro-textures on the flow dynamics and transient temperature field distributions at the friction interface. To explicitly distinguish between the experimental and computational approaches, the overall study structure and the employed methodologies are systematically summarized in the workflow diagram below (Figure 1).

### 2.1. Bionic Design Principle of the Regular Hexagonal Micro-Texture

**Figure 2:**
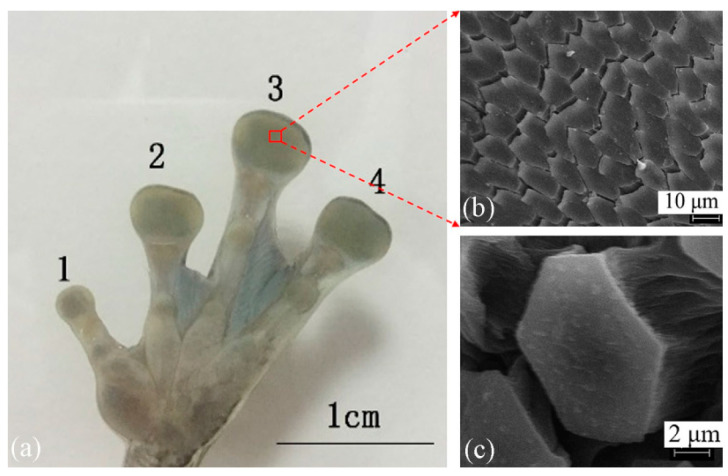
Structure of tree frog foot pad: (**a**) tree frog left fore foot (1—the first finger, 2—the second finger, 3—the third finger, 4—the fourth finger); (**b**) partial enlarged view of foot pad, magnified 1000 times; (**c**) single epithelial cell of foot pad, magnified 5000 times [43].

In nature, the tree frog foot pad can rapidly regulate the liquid content within its adhesive interface, ensuring stable adhesion [42]. This distinct capability for contact and friction control in wet environments is fundamentally attributed to the unique microstructural morphology of the pad surface, shaped by evolutionary processes. Microscopic observations (as shown in Figure 2) confirm that the epithelial tissue of the tree frog foot pad is densely populated with closely packed hexagonal columnar cells, delineated by an interconnected network of micro-scale channels [43]. Under wet conditions, this micro-morphology, consisting of hexagonal land regions and surrounding channels, demonstrates significant hydrodynamic advantages. The interwoven channel network acts as an efficient drainage system, swiftly expelling excess fluid from the contact interface. This action markedly reduces the hydrodynamic repulsion induced by fluid accumulation and promotes the establishment of solid contact between the pad and the substrate [39]. Inspired by the geometric configuration of this biological drainage microstructure, this study integrated a geometrically bio-inspired regular hexagonal micro-texture into the contact interface of a wet friction pair’s mating steel plate. By reshaping the three-dimensional geometric morphology of the friction interface, this approach aims to optimize both the fluid dynamic behavior and temperature field performance of the wet friction pair.

### 2.2. Bionic Micro-Texture Design and Parametric Study

To comprehensively investigate the micro-hydrodynamic mechanisms and transient thermal behaviors induced by the bio-inspired micro-textures, a dual-scale parametric design strategy was adopted. Two distinct geometric models were established: a pin-on-disc model dedicated to fundamental experimental validation, and a full-scale disc-on-disc model representing the macroscopic operating conditions of an actual wet clutch. This structural approach bridges microscopic physical verification with macroscopic industrial simulation.

#### 2.2.1. Bionic Micro-Texture Design and Parametric Study of Pin-on-Disc Friction Pair

**Figure 3:**
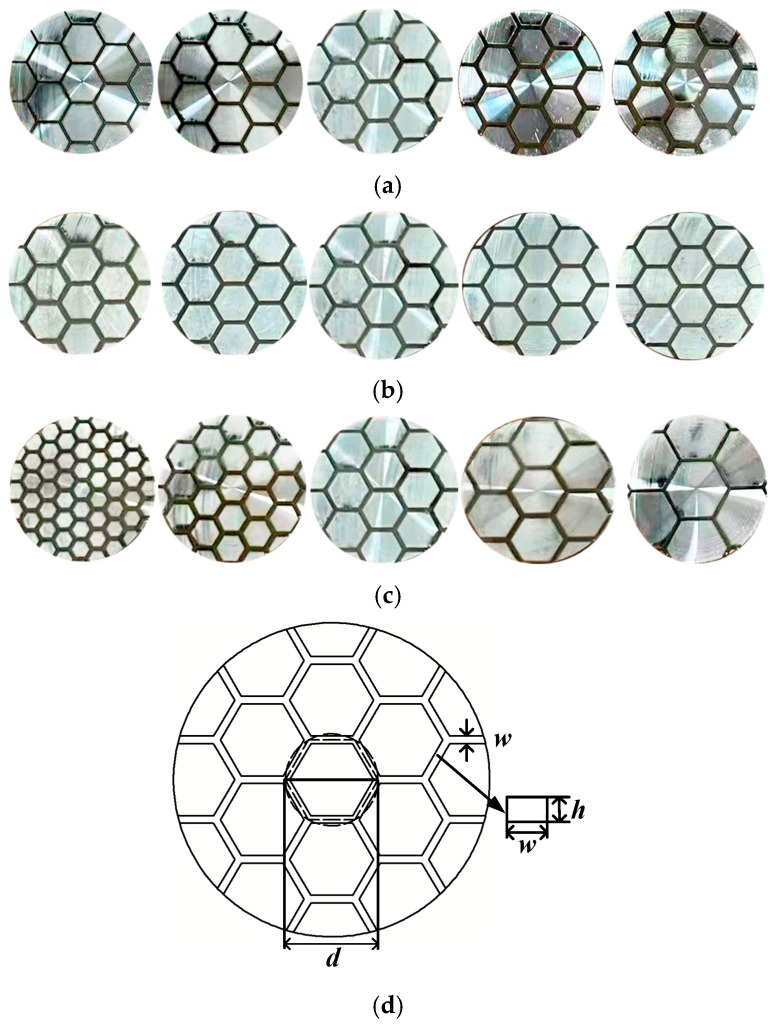
Schematics of the bionic hexagonal micro-textures with different structural parameters: (**a**) different groove widths; (**b**) different groove depths; (**c**) different circumcircle diameters; (**d**) definition of micro-textures’ structural parameters.

For the pin-on-disc friction pair, the bionic hexagonal micro-textures were parameterized on the contact surface of the pin, which simulates the mating steel plate. As illustrated in Figure 3, the topological variations in these micro-textures are strictly governed by three independent geometric variables: groove width (w), groove depth (h), and circumcircle diameter (d). To systematically assess their tribological effects, a comprehensive parametric matrix was formulated. The specific geometric ranges for these structural parameters are summarized in Table 1.

#### 2.2.2. Bionic Micro-Texture Design and Parametric Study of Disc-on-Disc Friction Pair

**Figure 4:**
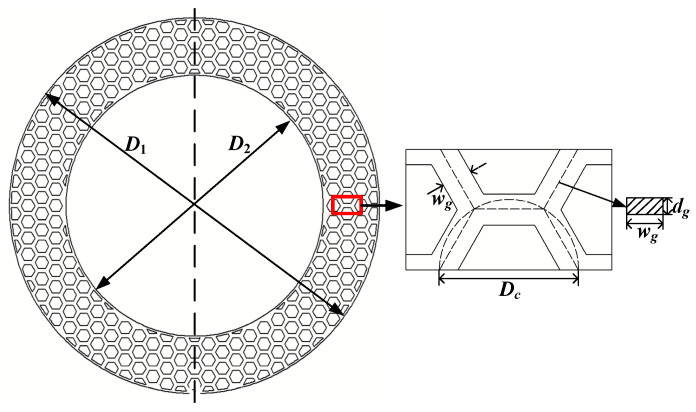
Schematic of the mating steel plate with bionic hexagonal micro-texture.

To explore the micro-hydrodynamic mechanisms of the disc-on-disc friction pair, bionic parameterization was subsequently applied to a full-size disc-on-disc friction pair. The macroscopic structural parameters of the disc-on-disc system and its corresponding bionic micro-textures are defined in Table 2. Figure 4 provides a parameterized schematic of the textured mating steel plate (taking $w_{g}=2.0\;mm$, $d_{g}=90\;\mu m$ and $D_{c}=9.0\;mm$ as an example). In this configuration, the overall dimensions of the mating steel plate are defined by an outer diameter ($D_{1}$) of 180 mm and an inner diameter ($D_{2}$) of 125 mm. Consistent with the pin-on-disc model, the geometric array of the micro-texture is continuously controlled by the groove width ($w_{g}$), groove depth ($d_{g}$), and circumcircle diameter ($D_{c}$).

### 2.3. Prototype Fabrication and Experimental Procedure

#### 2.3.1. Prototyping of Bionic Micro-Textures via Laser Surface Texturing

Based on the parametric design, the bionic hexagonal micro-textures were fabricated onto the bottom contact surface of the 45 steel pin using a precision laser surface texturing (LST) technique. Table 3 lists the specific processing parameters of the laser system employed. By strictly controlling these settings, a uniform surface roughness of Ra 2.2 was maintained inside the micro-textured grooves.

#### 2.3.2. Experimental Apparatus and Testing Procedure

To simulate the operational conditions of a wet clutch friction pair, pin-on-disc friction tests were executed using a UMT TriboLab tester (Bruker, San Jose, CA, USA). The pin specimen, designed to simulate the mating steel plate, was fabricated from 45 steel with a bottom surface roughness of Ra 1.6. The disc specimen, representing the friction plate, was composed of H62 brass with a surface roughness of Ra 3.2. Neither material underwent heat treatment during the machining process.

**Figure 5:**
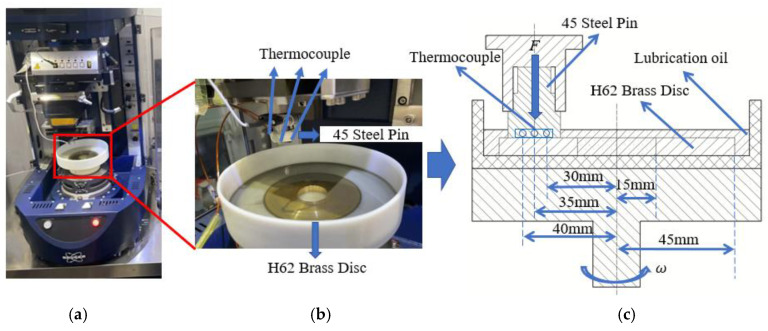
Schematic of the UMT Tribolab tester and the Pin-on-Disc testing module: (**a**) UMT TriboLab tester; (**b**) Pin-on-Disc testing module; (**c**) 2D schematic of the Pin-on-Disc testing module (the blue circles indicate the embedded positions of the thermocouples).

The pin was designed as a three-section stepped cylinder, presenting a 20 mm diameter contact face against the friction disc. To accurately record transient temperature fluctuations and map the radial temperature gradient, narrow holes were drilled into the pin’s substrate near the contact interface. Positioned radially at 30 mm, 35 mm, and 40 mm from the rotational axis, these holes were embedded with K-type thermocouples (measurement range 0–100 °C, accuracy ±0.1 °C; Yizhiding Precision Measurement & Control Co., Ltd., Yancheng, China). During the procedure, the disc specimen was secured to a rotating platform, while the pin specimen maintained sliding contact at an orbital radius of 35 mm. To properly simulate a wet friction environment, the entire friction pair was fully submerged in lubricant. A schematic representation of the experimental setup is provided in Figure 5 and the working condition parameters for the pin-on-disc experiment are summarized in Table 4.

Before the experiments, all specimens were ultrasonically cleaned in acetone and absolute ethanol for 10 min, subsequently rinsed with deionized water, and dried in a 60 °C vacuum oven. Once the experimental parameters were configured and the lubricant temperature reached stability, a predefined normal load was applied via a force sensor (measurement range 0–200 N, accuracy ±0.01 N; Bruker, San Jose, CA, USA), and the rotation was initiated. To ensure a stable mechanical contact regime, a 15 min running-in phase was completed for each pin prior to formal data acquisition.

### 2.4. CFD Simulation Model Setup

#### 2.4.1. CFD Model Assumptions and Boundary Conditions

To accurately analyze the localized fluid flow and temperature fields surrounding the bionic hexagonal micro-textures within the friction pair, this study developed and solved a fully 3D transient CFD model using ANSYS Fluent 2025 R1 [44]. Given that the CFD simulation model operated under standard atmospheric pressure and an ambient temperature of 30 °C while completely immersed in CD40 lubricant (Sinopec Lubricant Co., Ltd., Beijing, China), the numerical model incorporated the following rational assumptions and boundary conditions:

(1) The CD40 lubricant is treated as an incompressible Newtonian fluid with a temperature-dependent dynamic viscosity. Considering the upper structural portion of the steel pin is directly exposed to the ambient atmosphere during the pin-on-disc experimental validation process, the surrounding air is modeled as an ideal gas within the pin-on-disc CFD model.
(2) Given the atmospheric operating conditions and the absence of external force pumping, cavitation and two-phase flows are excluded; the fluid is strictly modeled as a continuous single-phase liquid.
(3) The fluid domain of the wet friction pair operates under a fully lubricated state. To satisfy momentum conservation, standard no-slip boundary conditions are enforced at all fluid–solid interfaces. The fluid velocity vectors (v) at the boundaries are prescribed to match the solid wall velocities:
  (1) v=0 (at the stationary mating steel plate)
  (2) v=ω×r  (at the rotating friction plate)
(4) The dominant frictional heat generated by the solid sliding motion is applied as a boundary heat flux at the fluid–solid interfaces, whereas the heat generated by viscous shearing is considered the internal heat source within the fluid domain. The conjugate heat transfer process at the interfaces is governed by Fourier’s law to ensure energy continuity:
  (3) $$-k_{f}\frac{\partial T_{f}}{\partial n}=-k_{s}\frac{\partial T_{s}}{\partial n}+q_{fric}$$
  where $k_{f}$ and $k_{s}$ denote the thermal conductivities of the fluid and solid respectively, $T_{f}$ and $T_{s}$ represent their local boundary temperatures, n is the unit normal vector pointing outward from the fluid domain, and $q_{fric}$ signifies the applied localized frictional heat flux.

It should be noted that the assumption of the fully lubricated regime within the fluid domain is rigorously justified based on the comprehensive measurement and mechanistic analysis of the coefficient of friction (COF) conducted in our previous pin-on-disc experimental research [11]. In the current study, the physical experiments are strictly utilized to provide transient thermal data for the verification of the CFD numerical model. The parameters of the CD40 lubricant utilized in this study are detailed in Table 5.

**Figure 6:**
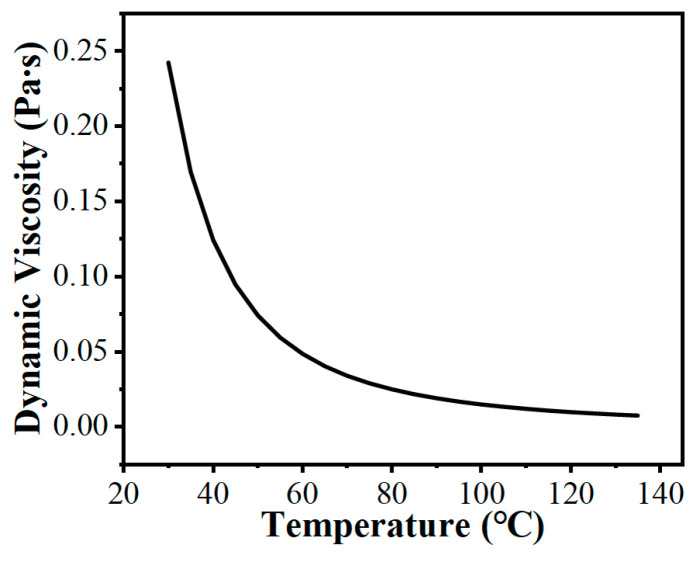
Dynamic viscosity–temperature curve of CD40 lubricant.

It is important to note that the dynamic viscosity of the CD40 lubricant is sensitive to temperature variations. To accurately capture the thermal–fluid behavior under high temperature rises during wet clutch friction pair operation, the dynamic viscosity is incorporated into the CFD model as a temperature-dependent variable. The specific dynamic viscosity–temperature curve is illustrated in Figure 6.

**Figure 7:**
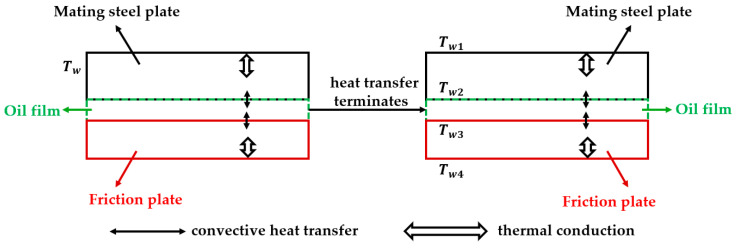
Schematic of the heat transfer mechanism across the fully lubricated interfaces.

Establishing the contact thermal resistance is essential for modeling the conjugate heat transfer across the interfaces. Based on the fundamental assumption that the wet friction pair operates under a fully lubricated state, the intervening continuous lubricating oil film separates the mating surfaces. The interfacial thermal resistance in the solid–fluid–solid domains is established, as shown in Figure 7. Based on material specifications corresponding to the pin-on-disc experiments, the parameters of the mating steel plate and the friction plate for both pin-on-disc friction pair and disc-on-disc friction pair are detailed in Table 6.

Under quasi-steady-state local convective heat transfer process, the macroscopic thermal transport dictated by Fourier’s law of heat conduction can be mathematically formulated as follows:

(4) $$\Phi =\frac{\left(T_{w1}-T_{w2}\right)Ak_{s-s}}{\delta_{1}}=\frac{\left(T_{w2}-T_{w3}\right)Ak_{f}}{\delta_{2}}=\frac{\left(T_{w3}-T_{w4}\right)Ak_{s-f}}{\delta_{3}}$$

where $\delta_{1}$, $\delta_{2}$ and $\delta_{3}$ denote their effective thicknesses; A is the contact area for heat transfer; and $T_{w1}$ through $T_{w4}$ denote the local interfacial boundary temperatures.

The temperature difference between the mating steel plate and the lower boundary of the oil film ($\Delta T_{1}$), as well as between the upper boundary of the oil film and the friction plate ($\Delta T_{2}$), can be obtained:

(5) $$\Delta T_{1}=T_{w1}-T_{w3}==\frac{\Phi }{A}\left(\frac{\delta_{1}}{k_{s-s}}+\frac{\delta_{2}}{k_{f}}\right)$$

(6) $$\Delta T_{2}=T_{w2}-T_{w4}=\frac{\Phi }{A}\left(\frac{\delta_{2}}{k_{f}}+\frac{\delta_{3}}{k_{s-f}}\right)$$

Based on this series thermal resistance network, the equivalent interfacial thermal resistance ($R_{s}$) between the mating steel plate and the fluid domain are defined as:

(7) $$R_{s}=\frac{\delta_{1}}{k_{s-s}}+\frac{\delta_{2}}{k_{f}}$$

Similarly, the equivalent interfacial thermal resistance ($R_{f}$) between the friction plate and the fluid domain are mathematically evaluated as:

(8) $$R_{f}=\frac{\delta_{2}}{k_{f}}+\frac{\delta_{3}}{k_{s-f}}$$

#### 2.4.2. CFD Simulation Model and Mesh Generation for the Pin-on-Disc Simulation

**Figure 8:**
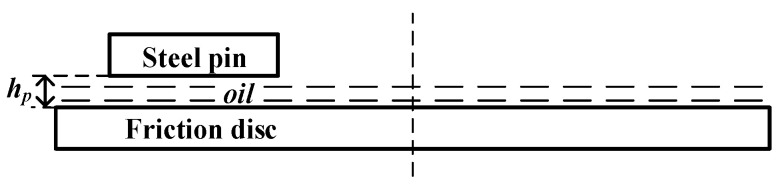
Schematic of the oil film thickness in the pin-on-disc friction pair.

The specific geometric dimensions of the pin-on-disc apparatus, along with the micro-texture parameters, were previously defined in Section 2.2. To guarantee the accuracy of the numerical analysis, the CFD simulation was built upon a rigorously defined fluid domain that accurately reflected the physical contact interface. In this model, the fluid domain was defined as the thin oil film confined between the lower, macroscopically smooth rotating brass disc (acting as the friction plate) and the upper stationary steel pin (acting as the mating steel plate). Since hydrodynamic performance was highly sensitive to clearance dimensions, the baseline oil film thickness was established at $h_{p}=20\;\mu m$ (as illustrated in Figure 8). The geometry of the hexagonal micro-texture was independently governed by groove width, depth, and circumcircle diameter.

**Figure 9:**
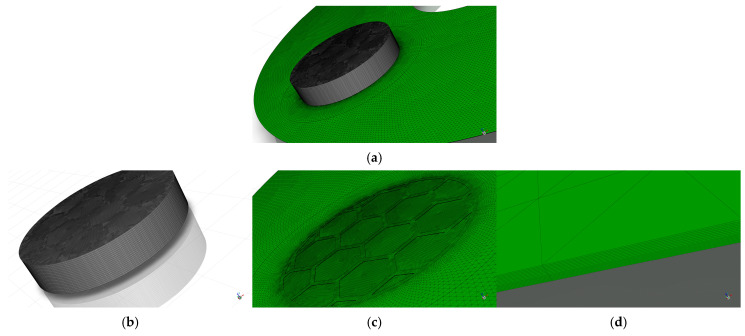
Schematic of the mesh generation for the pin-on-disc CFD simulation model: (**a**) overall computational domain mesh for the pin-on-disc model; (**b**) mesh of the solid domain; (**c**) mesh of the fluid domain; (**d**) local magnified mesh of the oil film.

To accurately capture the variations in fluid velocity and temperature gradients at the fluid–solid interface and within the micro-textures, high-quality computational meshes were generated for the fluid domain using ANSYS ICEM 2025 R1 (taking the bionic hexagonal micro-textures on the steel pin as an example, with structural parameters of w=0.5 mm, h=30 μm, and d=6.0 mm, as shown in Figure 9). Localized refinement and layers were applied near walls and inside the textured grooves to meet numerical precision standards. Because the micro-scale dimensions could cause automated meshing tools to produce excessively large element counts and poor mesh quality, ICEM was utilized to minimize round-off errors. To correctly simulate heat transfer and fluid shear, eight layers were assigned to the fluid side and five to the solid side. Coincident surfaces were then configured as coupled walls in Fluent.

In transient CFD analysis, numerical accuracy relies on both spatial (∆x) and temporal (∆t) discretization. To prevent errors typical of single-variable independence tests, a comprehensive grid and time-step independence study was executed [45,46]. By cross-combining four baseline mesh sizes ($∆x_{1}=0.8\;mm$, $∆x_{2}=0.6\;mm$, $∆x_{3}=0.4\;mm$, $∆x_{4}=0.2\;mm$) and four time steps ($∆t_{1}=0.01\;s$, $∆t_{2}=0.001\;s$, $∆t_{3}=0.0005\;s$, $∆t_{4}=0.0001\;s$), 16 distinct schemes were evaluated (as shown in Table 7). The final temperature ($T_{CH3}$) at a 40 mm radial location served as the monitoring variable. When the time step was reduced to $∆t_{2}$ and the mesh refined to $∆x_{3}$, the relative discrepancy remained strictly below 1.01%. Balancing accuracy with computational expense, Mesh 10 (∆ = 0.4 mm, $∆t_{2}=0.001\;s$) was adopted as the standard configuration, stabilizing the total cell count within the fluid domain at approximately 500,000.

#### 2.4.3. CFD Simulation Model and Mesh Generation for the Disc-on-Disc Simulation

**Figure 10:**
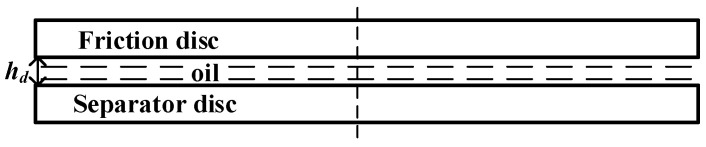
Schematic of the oil film thickness in the disc-on-disc friction pair.

**Figure 11:**
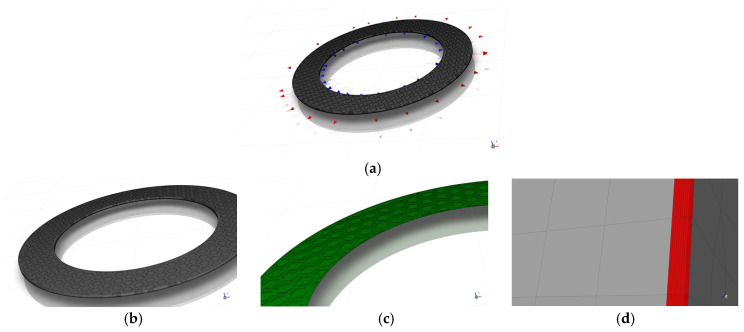
Schematic of the mesh generation for the disc-on-disc CFD simulation model: (**a**) overall computational domain mesh for the disc-on-disc model; (**b**) mesh of the solid domain; (**c**) mesh of the fluid domain; (**d**) local magnified mesh of the oil film.

The active fluid domain encompassed the oil film thickness ($h_{d}=25\;\mu m$) situated between the rotating friction plate (operating at 1000 rpm) and the stationary mating steel plate (as shown in Figure 10). Meshing procedures followed the identical protocols described in Section 2.4.2 (as shown in Figure 11). The grid independence verification similarly evaluated 16 schemes (as shown in Table 8), using the area-averaged outlet temperature ($T_{out}$) as the critical metric. Based on these tests, Mesh 10 was selected for the disc-on-disc analyses, stabilizing at roughly 4 million cells for the entire fluid volume.

#### 2.4.4. Numerical Solution Method

In rotating fluid systems, the determination of the flow regime (laminar or turbulent) typically depends on the Reynolds number (*Re*) [47]. In this study, the flow dynamics were dictated by the specific structural and operating parameters of pin-on-disc and disc-on-disc CFD configurations. Under the geometric dimensions and high-speed rotational conditions mentioned in Section 2.2.1, Section 2.2.2, Section 2.3.2 and Section 2.4.3, the calculated local *Re* values increase significantly along the radial direction, exceeding the critical transition threshold for laminar flow. This confirms that the fluid domains in both numerical models operate predominantly within the turbulent regime [48]. Given these factors, the RNG k-ε turbulence model was chosen to solve the governing equations. By incorporating an additional term in the dissipation rate equation, this model provided superior accuracy for swirling and vortex-dominated flows typical of grooved clutches [49]. The transport equations for the turbulent kinetic energy (*k*) and its dissipation rate (*ε*) are mathematically expressed as follows:

(9) $$\frac{\partial \left(\rho k\right)}{\partial t}+\frac{\partial \left(\rho ku_{i}\right)}{\partial x_{i}}=\frac{\partial }{\partial x_{j}}\left[\alpha_{k}\mu_{eff}\frac{\partial k}{\partial x_{j}}\right]+G_{k}+G_{b}-\rho \varepsilon -Y_{M}+S_{k}$$

(10) $$\frac{\partial \left(\rho \varepsilon \right)}{\partial t}+\frac{\partial \left(\rho \varepsilon u_{i}\right)}{\partial x_{i}}=\frac{\partial }{\partial x_{j}}\left[\alpha_{\varepsilon }\mu_{eff}\frac{\partial \varepsilon }{\partial x_{j}}\right]+C_{1\varepsilon }\frac{\varepsilon }{k}\left(G_{k}+C_{3\varepsilon }G_{b}\right)-C_{2\varepsilon }\rho \frac{\varepsilon^{2}}{k}-R_{\varepsilon }+S_{\varepsilon }$$

(11) $$R_{\varepsilon }=\frac{C_{\mu }\rho \eta^{3}\left(1-\eta /\eta_{0}\right)}{1+\beta \eta^{3}}\frac{\varepsilon^{2}}{k}$$

where ρ is the lubricant density, $u_{i}$ represents the velocity component, and $\mu_{eff}$ denotes the effective dynamic viscosity. The terms $\alpha_{k}$ and $\alpha_{\varepsilon }$ are the inverse effective Prandtl numbers for k and ε, respectively. $G_{k}$ and $G_{b}$ represent the generation of turbulent kinetic energy due to mean velocity gradients and buoyancy. The term $R_{\varepsilon }$ accounts for the effects of rapid strain and streamline curvature, which is crucial for accurately resolving the rotating flow fields in the friction pairs.

For pressure-velocity coupling, the SIMPLE algorithm was employed, which strictly enforced mass and momentum conservation for incompressible fluids [50]. To minimize numerical diffusion, all momentum, turbulence, and energy equations were discretized using a second-order upwind scheme. Based on independence test results, a fixed physical time step of ∆t=0.001 s was utilized. The pin-on-disc simulations were run for 60,000 time steps, while the disc-on-disc simulations ran for 1200 time steps, with a maximum of 20 iterations per step. Solutions were deemed converged when the scaled residuals dropped below $10^{-4}$ for continuity and momentum, and $10^{-6}$ for energy.

## 3. Results and Discussion

### 3.1. Accuracy Verification of the CFD Simulation Model Based on the Pin-on-Disc Experiment

To verify the reliability of the numerical approach, dynamic temperature rise data spanning 60 s were extracted from the pin-on-disc physical test (taking the bionic hexagonal micro-texture parameters w=0.5 mm, h=30 μm and d=6.0 mm at 30 N and 120 rpm as an example). These results were subsequently compared against the CFD predictions under equivalent boundary conditions.

**Figure 12:**
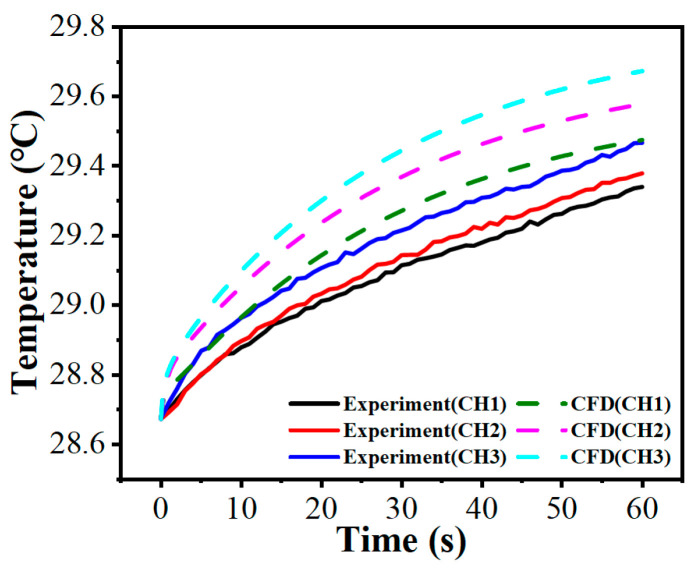
Comparison of temperature rise between the pin-on-disc experiment and the CFD simulation model.

To provide a rigorous and realistic validation, Root Mean Square Error (RMSE) and maximum absolute deviation were employed as the primary evaluation metrics. As shown in Figure 12, over the 60 s period, the maximum absolute deviation between the experimental data and CFD simulations are approximately 0.28 °C (observed at CH3). The overall RMSE for the three monitoring channels remains below 0.15 °C. These robust statistical metrics confirm the reliability of the established 3D transient CFD framework for subsequent analyses.

It is important to clarify the distinction between the data sources presented in this study. Figure 12 is the sole plot in this study that explicitly compares experimental measurements with computational results derived from the pin-on-disc CFD configuration. In Figure 12, the labels CH1, CH2, and CH3 denote the temperature monitoring channels located at radial distances of 30 mm, 35 mm, and 40 mm from the rotation center, respectively. Its primary purpose is strictly methodological validation. Building upon this verified numerical framework, it should be explicitly noted that all subsequent flow dynamics contours and transient temperature field plots presented in Section 3.2 are derived exclusively from the disc-on-disc CFD simulations.

### 3.2. Analysis of the Flow Field and Temperature Field Evolution Mechanisms of the Disc-on-Disc Friction Pair

#### 3.2.1. Analysis of Flow Dynamics

**Figure 13:**
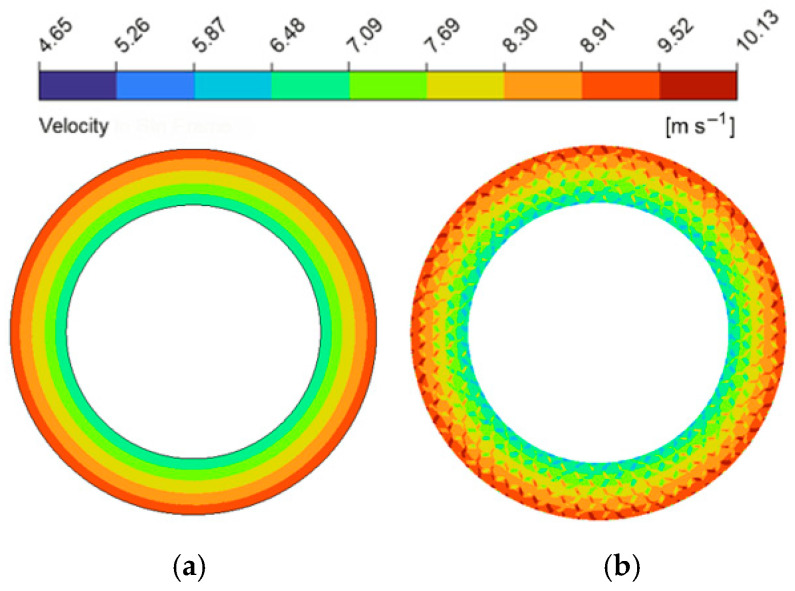
Comparison of flow velocity magnitude contour maps between the friction pair with bionic hexagonal micro-textures and the untextured friction pair at the $z=0.5h_{d}$ cross-section: (**a**) untextured friction pair; (**b**) textured friction pair.

The implementation of bionic micro-textures significantly influenced fluid distribution and hydrodynamic load-bearing properties. To quantify this, velocity magnitude contours at the mid-plane ($z=0.5h_{d}$) of the oil film were compared between a baseline textured pair (structural parameters of micro-texture: $w_{g}=2.0\;mm$, $d_{g}=90\;\mu m$ and $D_{c}=9.0\;mm$; these baseline parameters were utilized in all subsequent comparisons with the untextured friction pair) and an untextured pair (as shown in Figure 13). In the untextured friction pair, fluid motion is governed solely by viscous shear and centrifugal forces, resulting in a maximum localized velocity of 9.39 m/s. Following the addition of the micro-texture, this maximum velocity climbed to 10.13 m/s, representing a 7.9% increase. From the perspective of momentum conservation governed by the N-S equations, this localized acceleration can be theoretically explained. In an untextured friction pair, the fluid momentum is primarily balanced by uniform viscous shear and centrifugal forces. However, the geometric discontinuities introduced by the boundaries of the bionic micro-textures alter the local flow cross-section. According to the momentum balance equations, this geometric variation redistributes local viscous shear stresses and induces a localized spatial pressure gradient. To maintain momentum balance, this localized pressure gradient acts as an additional driving force that overcomes viscous resistance, consequently increasing the convective acceleration of the fluid elements and locally elevating the flow velocity [51]. Consequently, the average hydrodynamic pressure across the textured pair escalated to approximately 0.1 MPa (a 28% increase compared with the untextured pair’s 0.078 MPa).

**Figure 14:**
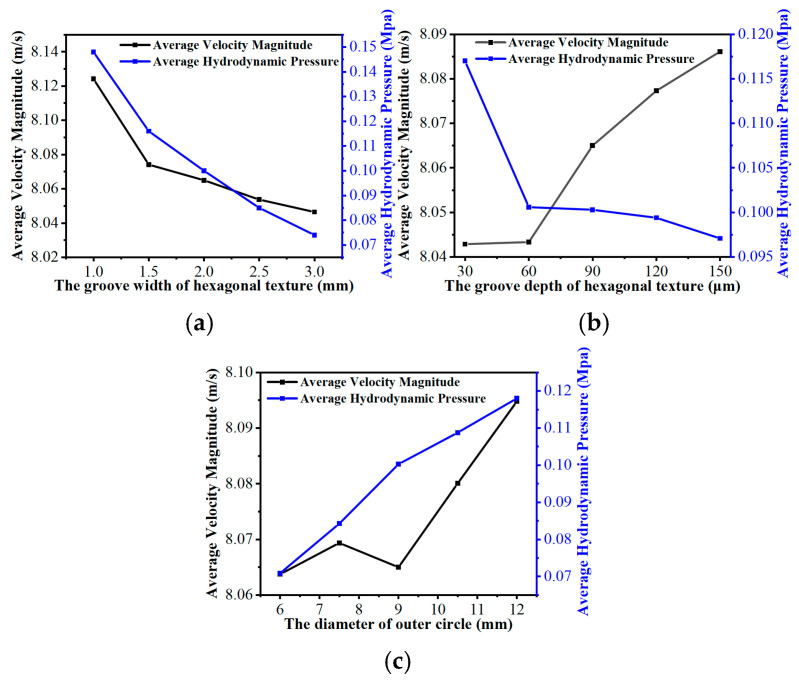
Variations in the average flow velocity and average fluid hydrodynamic pressure in the flow field with the structural parameters of the bionic hexagonal micro-texture: (**a**) groove width of the bionic hexagonal micro-texture; (**b**) groove depth of the bionic hexagonal micro-texture; (**c**) circumcircle diameter of the bionic hexagonal micro-texture.

**Figure 15:**
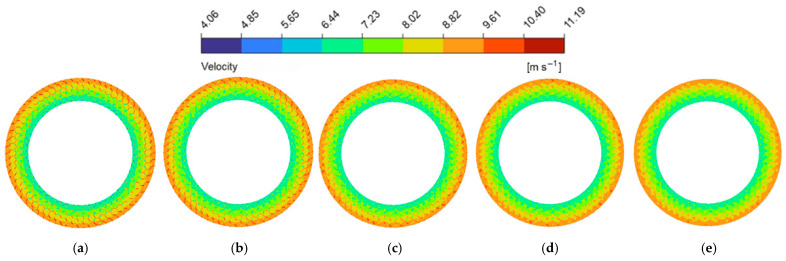
Flow velocity magnitude contour maps of the flow field under different groove widths of the bionic hexagonal micro-texture at the $z=0.5h_{d}$ cross-section: (**a**) $w_{g}=1.0\;mm$; (**b**) $w_{g}=1.5\;mm$; (**c**) $w_{g}=2.0\;mm$; (**d**) $w_{g}=2.5\;mm$; (**e**) $w_{g}=3.0\;mm$.

**Figure 16:**
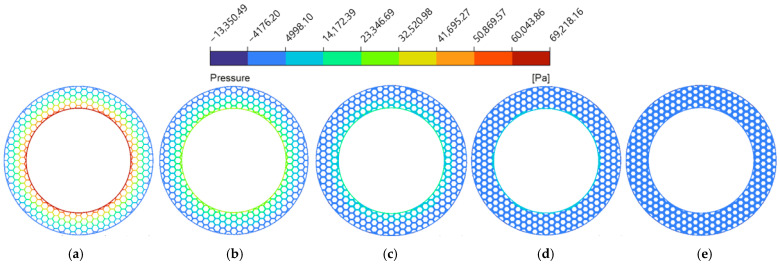
Hydrodynamic pressure magnitude contour maps of the flow field under different groove widths of the bionic hexagonal micro-texture at the $z=0.5d_{g}$ cross-section: (**a**) $w_{g}=1.0\;mm$; (**b**) $w_{g}=1.5\;mm$; (**c**) $w_{g}=2.0\;mm$; (**d**) $w_{g}=2.5\;mm$; (**e**) $w_{g}=3.0\;mm$.

**Figure 17:**
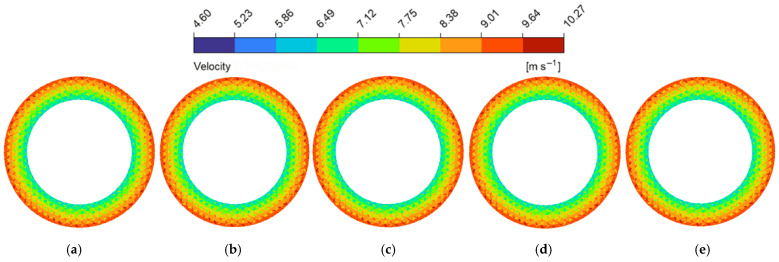
Flow velocity magnitude contour maps of the flow field under different groove depths of the bionic hexagonal micro-texture at the $z=0.5h_{d}$ cross-section: (**a**) $d_{g}=30\;\mu m$; (**b**) $d_{g}=60\;\mu m$; (**c**) $d_{g}=90\;\mu m$; (**d**) $d_{g}=120\;\mu m$; (**e**) $d_{g}=150\;\mu m$.

**Figure 18:**
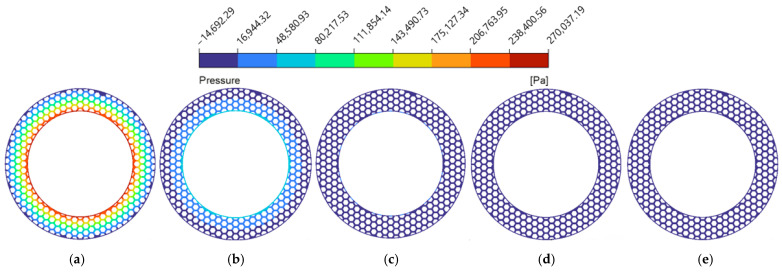
Hydrodynamic pressure magnitude contour maps of the flow field under different groove depths of the bionic hexagonal micro-texture at the $z=0.5d_{g}$ cross-section: (**a**) $d_{g}=30\;\mu m$; (**b**) $d_{g}=60\;\mu m$; (**c**) $d_{g}=90\;\mu m$; (**d**) $d_{g}=120\;\mu m$; (**e**) $d_{g}=150\;\mu m$.

**Figure 19:**
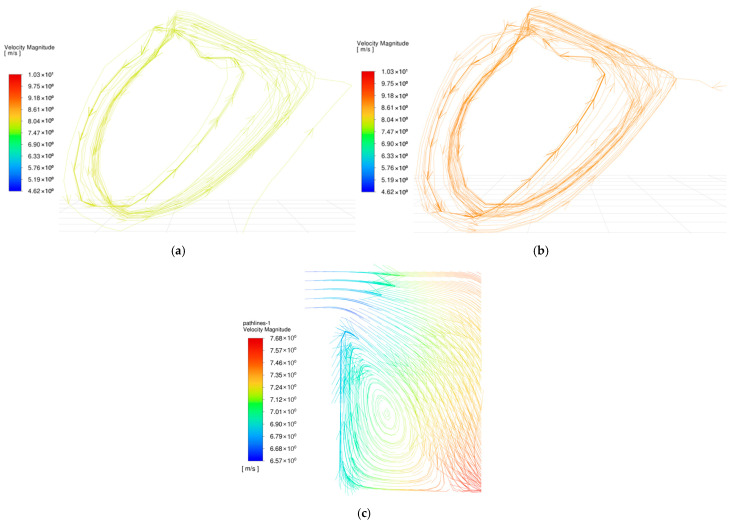
Fluid streamline distributions and indicative velocity vectors within the bionic hexagonal micro-texture at the $z=0.5h_{d}$ cross-section for $d_{g}=90\;\mu m$: (**a**) flow field in the xy-plane at the land region edge (lower-velocity region); (**b**) flow field in the xy-plane at the land region edge (higher-velocity region); (**c**) cross-sectional flow field in the yz-plane inside the groove.

**Figure 20:**
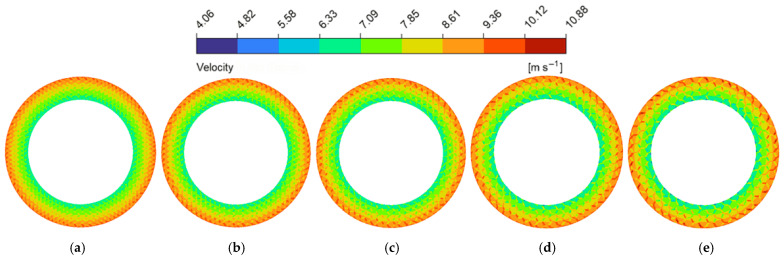
Flow velocity magnitude contour maps of the flow field under different circumcircle diameters of the bionic hexagonal micro-texture at the $z=0.5h_{d}$ cross-section: (**a**) $D_{c}=6.0\;mm$; (**b**)$\;D_{c}=7.5\;mm$; (**c**)$\;D_{c}=9.0\;mm$; (**d**)$\;D_{c}=10.5\;mm$; (**e**)$\;D_{c}=12.0\;mm$.

**Figure 21:**
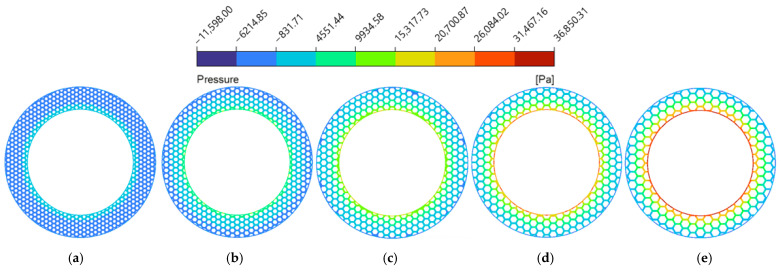
Hydrodynamic pressure magnitude contour maps of the flow field under different circumcircle diameters of the bionic hexagonal micro-texture at the $z=0.5d_{g}$ cross-section: (**a**) $D_{c}=6.0\;mm$; (**b**)$\;D_{c}=7.5\;mm$; (**c**)$\;D_{c}=9.0\;mm$; (**d**)$\;D_{c}=10.5\;mm$; (**e**)$\;D_{c}=12.0\;mm$.

Further investigations explored how specific micro-texture geometries nonlinearly dictated flow dynamics (as shown in Figure 14 and visually detailed in Figure 15, Figure 16, Figure 17, Figure 18, Figure 19, Figure 20 and Figure 21). And it is noteworthy that a magnitude of negative pressure is observed in the subsequent hydrodynamic pressure contours (e.g., Figure 16, Figure 18 and Figure 21). This is because when the lubricant flows through grooves, there are obvious convergent and divergent areas which lead to pressure value changes from positive to negative. As the fluid moves from the land regions into the deeper grooves, the flow channel diverges, which generates a rapid drop in localized hydrodynamic pressure.

As the groove width ($w_{g}$) expanded from 1.0 mm to 3.0 mm (Figure 14a and Figure 15), both average velocity and average hydrodynamic pressure exhibited monotonic declines. This is because the wider grooves provide a more unobstructed flow channel for the lubricant, which exacerbates the side leakage of the fluid in both the circumferential and radial directions. Consequently, the overall shear resistance of the oil film increases, and the internal squeezing effect decreases [52]. This phenomenon is explicitly corroborated by the hydrodynamic pressure contour maps at the $z=0.5d_{g}$ cross-section (Figure 16). The contours intuitively demonstrate that wider grooves progressively dissipate the localized high-pressure concentrations at the boundaries of the land regions. As a result, the average fluid hydrodynamic pressure dropped from 0.15 MPa to 0.07 MPa, reducing the fluid’s flow potential energy and consequently slowing the average flow velocity.

Conversely, modifying the groove depth ($d_{g}$) produced distinct nonlinear trends (as shown in Figure 14b). Deeper grooves enlarge the fluid volume, which continuously reduces the shearing effect imposed by land regions of micro-textures. This is visually confirmed by the hydrodynamic pressure contour maps at the $z=0.5d_{g}$ cross-sections (Figure 18), where the progressive deepening of the grooves alleviates the internal fluid squeezing action from the upper boundary, leading to a steady decrease in average hydrodynamic pressure. However, despite this pressure reduction, the average flow velocity exhibited a continuous upward trend. As illustrated in the velocity contour maps (Figure 17), localized high-velocity zones emerged at the boundaries between grooves and texture lands. For depths of $d_{g}\geq 90\;\mu m$, the deeper grooves store additional fluid for secondary lubrication [53]. The bulk fluid experiences flow separation as it travels over the land regions of micro-textures, triggering vortices at the edges of the land regions [54] (as shown in Figure 19). These micro-vortices act as internal drivers, propelling fluid rapidly within the grooves and raising the global average velocity.

Increasing the circumcircle diameter ($D_{c}$) led to a steady rise in hydrodynamic pressure (as shown in Figure 14c), while flow velocity initially fluctuated and then rose significantly. The hydrodynamic pressure contour maps at the $z=0.5d_{g}$ cross-section (Figure 21) reveal the underlying mechanism: larger $D_{c}$ values geometrically extend the continuous solid area of the individual land regions. This extension provides a sufficient spatial domain for the micro-hydrodynamic wedging effect to establish, which intensifies the localized hydrodynamic pressure peaks and elevates the overall average hydrodynamic pressure. Regarding the flow velocity, as shown in Figure 20, smaller $D_{c}$ values corresponded to a denser micro-texture array, which increased the number of land regions of the micro-textures and forced the fluid to undergo higher viscous dissipation, leading to shrinkage of the high-velocity zones. However, as $D_{c}$ reached 10.5 mm and 12.0 mm (as shown in Figure 20d,e), the spatial density decreased, and the radial length of the grooves increased. During the flow process, the fluid reduces its kinetic energy consumption, thereby accelerating the flow velocity.

#### 3.2.2. Evolution Characteristics of the Average Bulk Temperature of the Friction Pair

**Figure 22:**
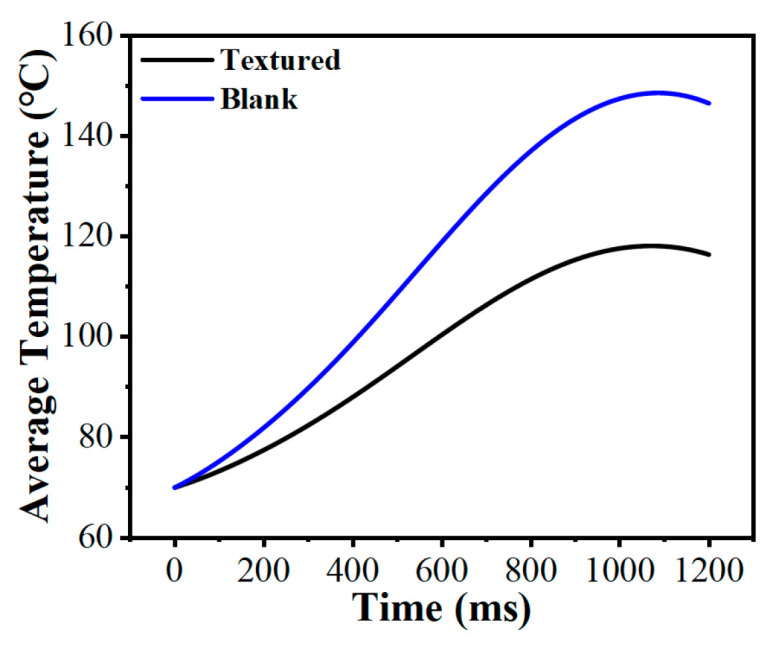
Comparison of the average bulk temperature between the friction pair with bionic hexagonal micro-textures and the untextured friction pair.

The average bulk temperature is a direct indicator of the macroscopic thermal balance within the friction pair. Figure 22 contrasts the transient bulk temperature evolution over a 1200 ms operating cycle. Lacking internal grooves for lubricant circulation, the untextured friction pair experienced severe heat accumulation, causing its average bulk temperature to climb nonlinearly to 146.5 °C. In contrast, the micro-textured friction pair successfully suppressed this accumulation; after an initial rise, the curve flattened, peaking at roughly 116.4 °C. This represented a 20.5% reduction in the maximum average bulk temperature. The interconnected grooves not only trap more lubricant but also expand the effective convective heat transfer area at the solid–liquid interface [55], effectively alleviating the accumulation of heat.

**Figure 23:**
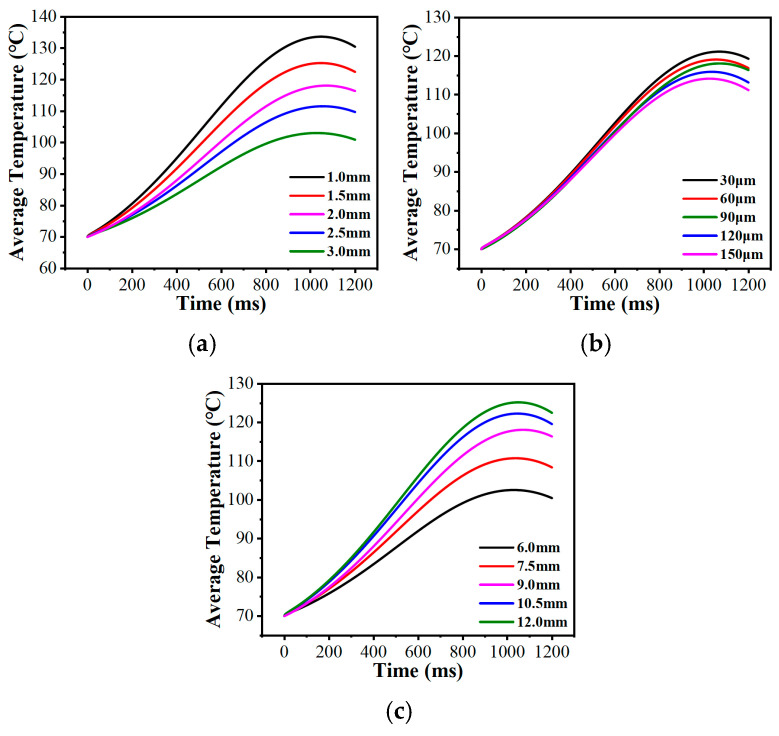
Variations in the average bulk temperature with the structural parameters of the bionic hexagonal micro-texture: (**a**) groove width of the bionic hexagonal micro-texture; (**b**) groove depth of the bionic hexagonal micro-texture; (**c**) circumcircle diameter of the bionic hexagonal micro-texture.

Adjusting the micro-texture’s internal dimensions fine-tuned the average bulk temperature of the friction pair. Figure 23a,b demonstrate that enlarging either the groove width ($w_{g}$) or depth ($d_{g}$) lowered the average bulk temperature. Wider and deeper grooves hold a larger volume of lubricant and increases the area extension factor of the interface, which consequently yields higher heat transfer efficiency through the combination of the lubricant and the local fluid within the grooves [56]. This enables the convective heat transfer process of the system to reach dynamic equilibrium earlier, thereby effectively lowering the peak of the bulk average temperature curve. Conversely, increasing the circumcircle diameter ($D_{c}$) tended to elevate the average bulk temperature (Figure 23c). A larger $D_{c}$ elongates the individual land region of micro-texture, thereby increasing the actual contact area responsible for frictional heat generation [57], which pushes the macroscopic temperature higher.

#### 3.2.3. Spatial Fluctuation Analysis of the Radial Temperature Gradient

**Figure 24:**
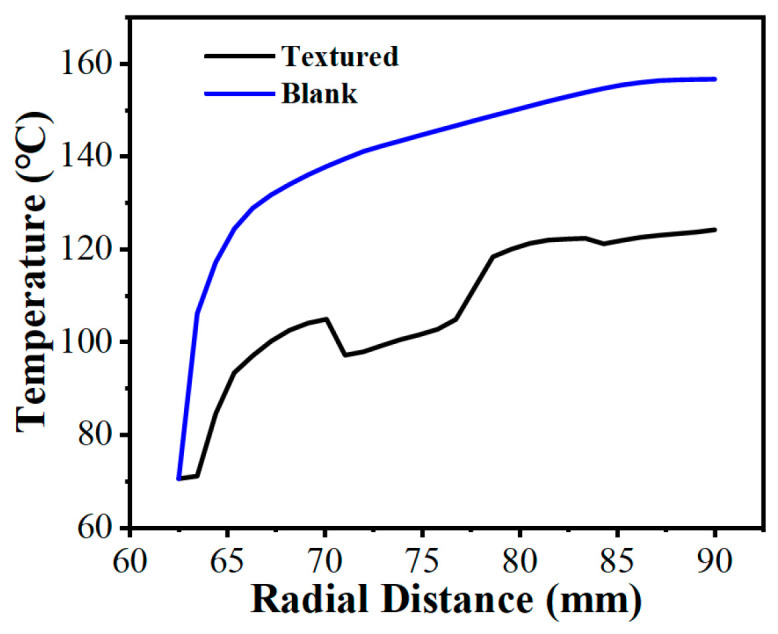
Comparison of radial temperature gradient variations between the friction pair with bionic hexagonal micro-textures and the untextured friction pair at the $z=0.5h_{d}$ cross-section from a radius of 62.5 mm to 90 mm.

**Figure 25:**
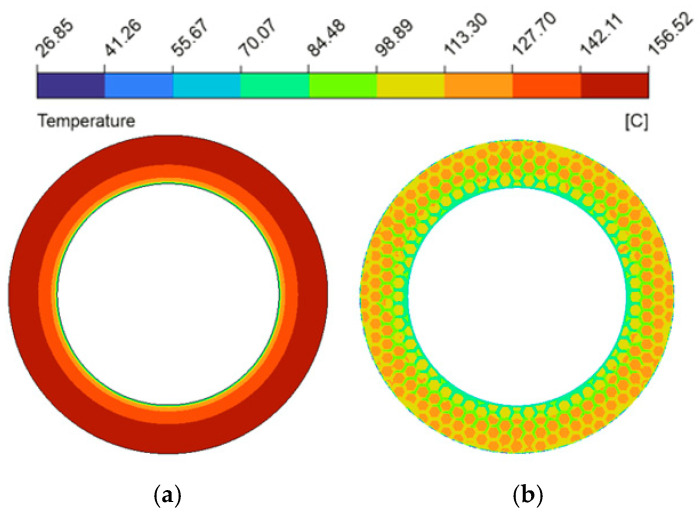
Comparison of temperature magnitude contour maps between the friction pair with bionic hexagonal micro-textures and the untextured friction pair: (**a**) untextured friction pair; (**b**) textured friction pair at the $z=0.5h_{d}$ cross-section.

The spatial uniformity of the radial temperature gradient is a vital metric for predicting thermo-hydrodynamic performance. Severe spatial temperature gradients can cause extreme non-uniformity in the localized thermal field [58], leading to drops in lubricant viscosity that destabilize the hydrodynamic oil film and degrade the overall tribological performance. Figure 24 plotted the radial temperature distribution at the fluid mid-plane ($z=0.5h_{d}$) of the oil film under thermal equilibrium (from a radius of 62.5 mm to 90 mm). Because heat generation scaled with the square of the local linear velocity [59], temperatures in both configurations trended upward toward the outer edge. In the untextured pair, uninterrupted heat transferred along the smooth interface, causing the temperature to reach 156.7 °C at the periphery. This macroscopic thermal accumulation is visually corroborated by the spatial temperature contour map (Figure 25a), which displays a widespread and continuous severe high-temperature annular band. However, the bionic micro-texture limited the maximum radial temperature to 124.2 °C (a 20.7% reduction). The corresponding temperature contour map (Figure 25b) illustrates that the interconnected grooves effectively partition the contiguous thermal field, utilizing the cooler retained lubricant to disrupt localized heat accumulation. Furthermore, the micro-textures fundamentally altered the temperature profile, changing it from a continuous curve to a fluctuating pattern characterized by alternating step-like climbs and localized cooling troughs, which profoundly mitigated the radial temperature gradients.

**Figure 26:**
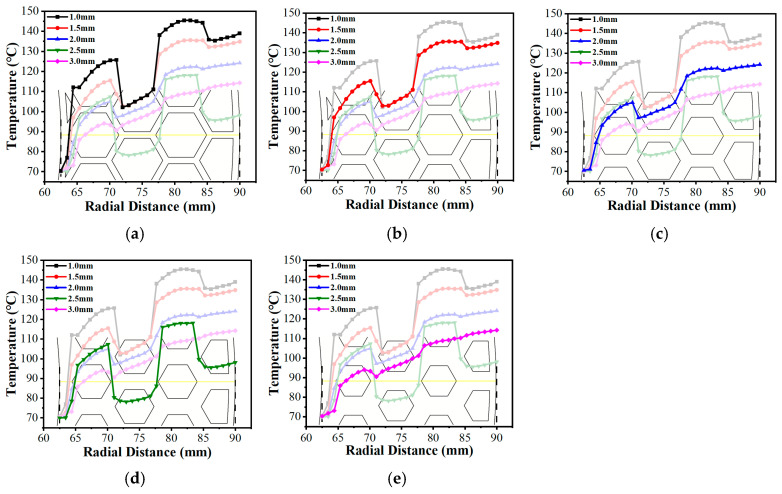
Variations in the radial temperature gradient under different groove widths of the bionic hexagonal micro-texture at the $z=0.5h_{d}$ cross-section from a radius of 62.5 mm to 90 mm (the yellow solid line represents the extraction path of the radial temperature distribution): (**a**) $w_{g}=1.0\;mm$; (**b**) $w_{g}=1.5\;mm$; (**c**) $w_{g}=2.0\;mm$; (**d**) $w_{g}=2.5\;mm$; (**e**) $w_{g}=3.0\;mm$.

**Figure 27:**
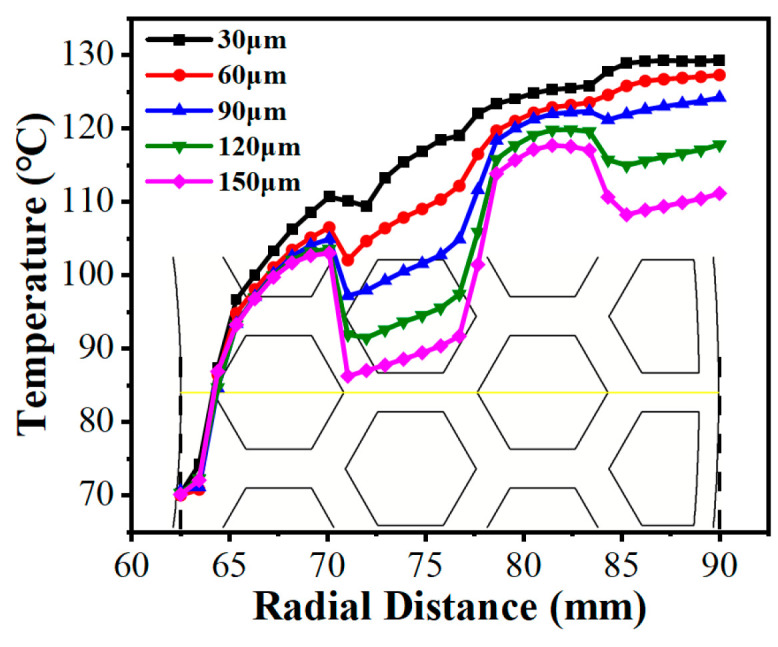
Variations in the radial temperature gradient under different groove depths of the bionic hexagonal micro-texture at the $z=0.5h_{d}$ cross-section from a radius of 62.5 mm to 90 mm (the yellow solid line represents the extraction path of the radial temperature distribution).

**Figure 28:**
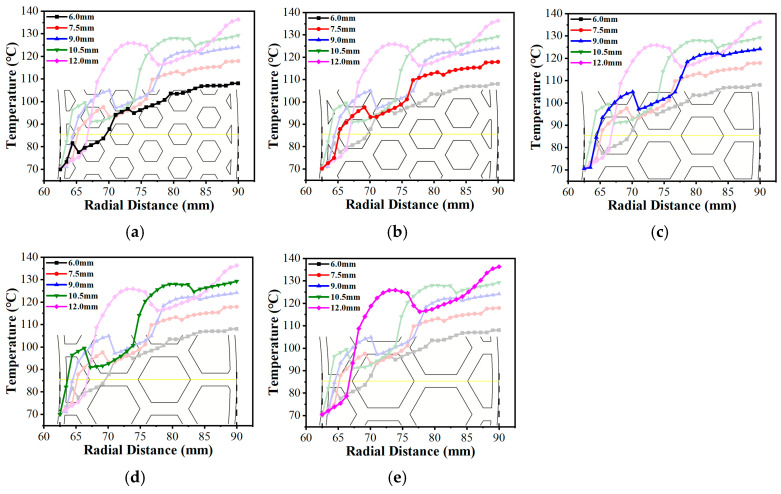
Variations in the radial temperature gradient under different circumcircle diameters of the bionic hexagonal micro-texture at the $z=0.5h_{d}$ cross-section from a radius of 62.5 mm to 90 mm : (**a**) $D_{c}=6.0\;mm$; (**b**)$\;D_{c}=7.5\;mm$; (**c**)$\;D_{c}=9.0\;mm$; (**d**)$\;D_{c}=10.5\;mm$; (**e**)$\;D_{c}=12.0\;mm$.

To clarify this fluctuating mechanism, the radial temperature profile was superimposed onto the 2D spatial layout of the micro-textures (as shown in Figure 26, Figure 27 and Figure 28). A strict phase alignment existed: temperature peaks matched the physical locations of the land regions of micro-texture, where frictional heating was direct and intense. Conversely, temperature troughs aligned precisely with the grooves, where the presence of cooler lubricant facilitated convective heat transfer and disrupted continuous heat accumulation [60].

**Figure 29:**
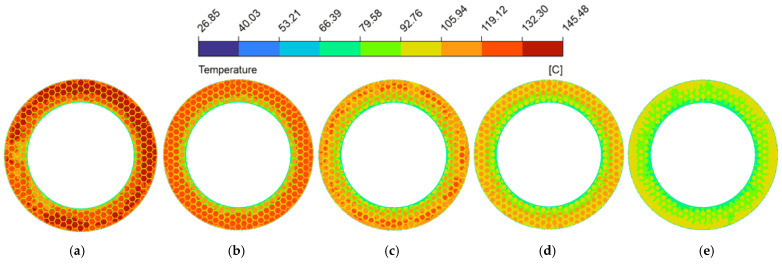
Temperature magnitude contour maps under different groove widths of the bionic hexagonal micro-texture at the $z=0.5h_{d}$ cross-section from a radius of 62.5 mm to 90 mm: (**a**) $w_{g}=1.0\;mm$; (**b**) $w_{g}=1.5\;mm$; (**c**) $w_{g}=2.0\;mm$; (**d**) $w_{g}=2.5\;mm$; (**e**) $w_{g}=3.0\;mm$.

Analyzing the structural parameters further revealed that as groove width ($w_{g}$) increased from 1.0 mm to 2.5 mm (as shown in Figure 26), the expanded channels accommodated a larger volume of low-temperature lubricant. The corresponding temperature contour maps (Figure 29a–d) intuitively capture this effect, displaying a progressive fading and shrinkage of the high-temperature patches on the land regions. This confirms the strengthened localized convective heat transfer, which gradually reduces the overall temperature level. However, excessively wide grooves ($w_{g}=3.0\;mm$, Figure 29e) induced expansive low-velocity zones as previously established in Figure 15e, which weakened the convective heat transfer capacity of the fluid, consequently leading to an elevation of the radial temperature gradient.

**Figure 30:**
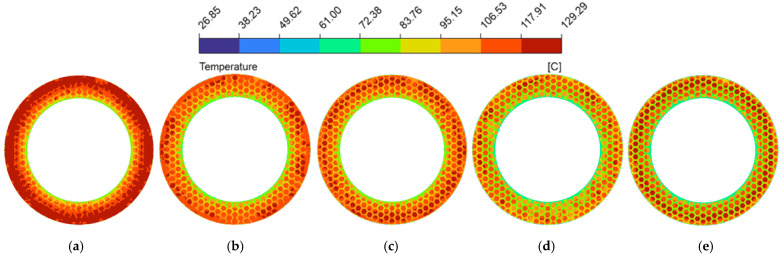
Temperature magnitude contour maps under different groove depths of the bionic hexagonal micro-texture at the $z=0.5h_{d}$ cross-section from a radius of 62.5 mm to 90 mm: (**a**) $d_{g}=30\;\mu m$; (**b**) $d_{g}=60\;\mu m$; (**c**) $d_{g}=90\;\mu m$; (**d**) $d_{g}=120\;\mu m$; (**e**) $d_{g}=150\;\mu m$.

Figure 27 and the corresponding temperature contour maps (Figure 30) demonstrated that deeper grooves ($d_{g}$) consistently lowered the radial temperature gradient. The progressive deepening of the grooves dissolves the severe thermal clusters across the contour plots. The vortices induced at the edges of the land regions ($d_{g}\geq 90\;\mu m$) actively promote the heat exchange between the fluid at the bottom of the groove and the upper friction interface [10], thereby effectively suppressing the emergence of high-temperature peaks and promoting the decline of the overall temperature gradient. It is worth noting that when the micro-texture reached a deeper groove depth ($d_{g}\geq 90\;\mu m$), the radial temperature curve exhibited a trend of significantly slowed temperature rise or even localized decrease at the edge of the plateau region; this was similarly caused by the vortex effect excitation at this location.

**Figure 31:**
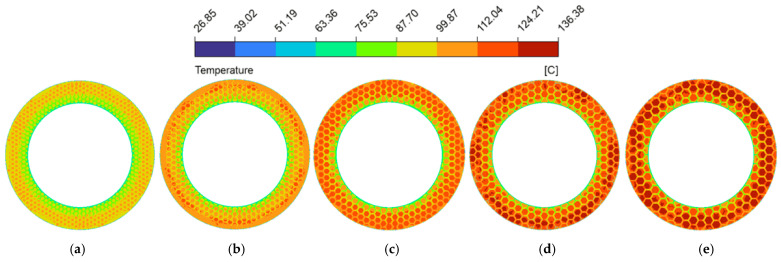
Temperature magnitude contour maps under different circumcircle diameters of the bionic hexagonal micro-texture at the $z=0.5h_{d}$ cross-section from a radius of 62.5 mm to 90 mm: (**a**) $D_{c}=6.0\;mm$; (**b**)$\;D_{c}=7.5\;mm$; (**c**)$\;D_{c}=9.0\;mm$; (**d**)$\;D_{c}=10.5\;mm$; (**e**)$\;D_{c}=12.0\;mm$.

Because altering the circumcircle diameter ($D_{c}$) directly adjusted the arrangement density of the micro-texture grid, the actual physical location where the fixed radial sampling line intersects the grooves changed, leading to a distinct lateral translation of the “troughs” on the curve (as shown in Figure 28). More importantly, as the circumcircle diameter increased, both the spatial temperature contour maps (Figure 31) and the radial temperature gradients exhibited a continuous upward trend. This phenomenon occurs because larger $D_{c}$ elongated the individual land regions. The temperature contour maps display these extended geometric domains evolving into broader, more intense high-temperature patches, as the fluid must flow across a longer distance of the frictional heat generation zone before encountering the next groove. This subsequently weakens the convective heat transfer effect between the hot and cold fluids, resulting in continuous heat accumulation. Consequently, a longer continuous temperature rise stage appeared on the temperature curve, and the proportion of the trough regions on the curve gradually decreased.

## 4. Conclusions

Focusing on the flow characteristics and temperature field of the wet clutch friction pair, this study developed 3D transient CFD models incorporating bionic regular hexagonal micro-textures on the mating steel plate. The pin-on-disc experiments were utilized strictly to validate the accuracy and reliability of the numerical algorithms within the pin-on-disc CFD model. Upon successful methodological verification, this numerical framework was extrapolated to a full-scale disc-on-disc CFD model to analyze the evolution mechanisms of its micro-flow dynamics and transient temperature fields. Based on the disc-on-disc simulations, the following primary conclusions are drawn:

(1) The introduction of tree-frog-inspired bionic micro-textures onto the surface of the mating steel plate significantly optimized interfacial fluid dynamics. Compared to untextured surfaces, the micro-textures increased the local maximum flow velocity by 7.9%. The interconnected groove network expanded the effective convective heat transfer area, actively limiting transient heat accumulation. This reduced the maximum average bulk temperature and the maximum radial temperature by 20.5% and 20.7%, respectively, enhancing the system’s thermo-hydrodynamic performance.
(2) Modifying structural parameters of the micro-texture directly dictated micro-flow behavior. Widening the grooves increased lateral fluid leakage, which decreased both the average flow velocity and the hydrodynamic pressure. Deepening the grooves similarly reduced pressure but uniquely promoted vortices at the land edges (when $d_{g}\geq 90\;\mu m$), which served to increase the local average flow velocity. Enlarging the circumcircle diameter limited kinetic energy losses and increased hydrodynamic pressure, facilitating higher fluid velocities.
(3) The micro-textures stabilized the overall radial temperature gradient, converting the continuously rising thermal profile into an alternating step-like distribution. When the fluid flowed over the land regions, a temperature rise peak was triggered due to frictional heat generation. Upon entering the groove region, convective heat transfer between the hot and cold fluids was promoted, consequently forming a cooling trough.
(4) Moderately increasing groove width and depth expanded the fluid volume available for convective heat transfer and improved the interface’s effective heat transfer area, effectively lowering the average bulk temperature. However, excessive widening (e.g., $w_{g}=3.0\;mm$) created low-velocity flow regions, which caused an overall elevation in the radial temperature gradient. Similarly, expanding the circumcircle diameter excessively extended the heat-generating land regions, which compromised the frequency of convective heat exchange and subsequently raised both the average bulk temperature and the overall radial temperature gradient.

While the wet friction pair consisting of a friction plate and a mating steel plate featuring bionic hexagonal micro-texture exhibits enhanced thermo-hydrodynamic performance compared to untextured, its transition to wet clutch systems requires further consideration of manufacturing constraints. Currently, the fabrication of these precise micro-textures relies primarily on laser surface texturing. Although LST provides necessary precision for laboratory prototyping, it is inherently characterized by high time costs and limited processing efficiency, restricting its scalability for mass production. To enhance the overall industrial feasibility, future research will focus on utilizing ultra-precision CNC milling to fabricate the hexagonal micro-textures on the mating steel plate. This manufacturing methodology is anticipated to improve scalability and ensure the necessary robustness of the friction pairs under the demanding operational conditions of transmissions.

## Figures and Tables

**Table 1:** Geometric parameters of the pin-on-disc friction pair for the bionic hexagonal micro-texture on the steel pin.

Parameters		Values				
Geometric parameters of the bionic hexagonal micro-texture	Groove depth (h/μm)	30	60	90	120	150
	Circumcircle diameter (d/mm)	3.0	4.5	6.0	7.5	9.0
	Groove width (w/mm)	0.3	0.4	0.5	0.6	0.7

**Table 2:** Geometric parameters of the disc-on-disc friction pair and value ranges of the structural parameters for the bionic hexagonal micro-texture on the mating steel plate.

Parameters		Values				
Outer diameter of the mating steel plate ($D_{1}/mm$)		180				
Inner diameter of the mating steel plate ($D_{2}/mm$)		125				
Geometric parameters of the bionic hexagonal micro-texture	Groove depth ($d_{g}/\mu m$)	30	60	90	120	150
	Circumcircle diameter ($D_{c}/mm$)	6.0	7.5	9.0	10.5	12.0
	Groove width ($w_{g}/mm$)	1.0	1.5	2.0	2.5	3.0

**Table 3:** Parameters of laser processing.

Parameters	Values
Laser power (W)	10
Wave length (nm)	1064
Scanning speed (mm/s)	500
Pulse duration (ns)	100
Diameter of defocused laser (mm)	0.05
Number of scans	2

**Table 4:** Operating parameters of pin-on-disc experiment.

Parameters		Values				
Operating parameters	Normal load (F/N)	30	35	40	45	50
	Rotational speed (ω/rpm)	130	150	170	190	210

**Table 5:** Parameters of CD40 lubricant.

Parameters	Values	Unit
Density (ρ/30°C)	880	$kg/m^{3}$
Dynamic viscosity (μ/30 °C)	0.243	Pa·s
Dynamic viscosity (μ/100 °C)	0.012	Pa·s
Specific heat capacity ($c_{p}$)	1900	J/(kg·°C)
Thermal conductivity ($k_{f}$)	0.144	W/(m·°C)

**Table 6:** Parameters of the mating steel plate and the friction plate.

Types	Parameters	Values	Unit
Mating steel plate	Density (ρ)	7850	$kg/m^{3}$
	Specific heat capacity ($c_{p-s}$)	460	J/(kg·°C)
	Thermal conductivity ($k_{s-s}$)	48	W/(m·°C)
Friction plate	Density (ρ)	8500	$kg/m^{3}$
	Specific heat capacity ($c_{p-f}$)	385	J/(kg·°C)
	Thermal conductivity ($k_{s-f}$)	109	W/(m·°C)

**Table 7:** Grid and time-step independence test for the CFD simulation model of the pin-on-disc friction pair.

Mesh	∆x	∆t	$T_{CH3(i,j)}/{}^{\circ}C$	$\varepsilon_{p}$	Mesh	∆x	∆t	$T_{CH3(i,j)}/{}^{\circ}C$	$\varepsilon_{p}$
1	$∆x_{1}$	$∆t_{1}$	30.593	3.91	9	$∆x_{3}$	$∆t_{1}$	30.099	2.23
2	$∆x_{1}$	$∆t_{2}$	30.473	3.50	10	$∆x_{3}$	$∆t_{2}$	29.745	1.03
3	$∆x_{1}$	$∆t_{3}$	30.087	2.19	11	$∆x_{3}$	$∆t_{3}$	29.710	0.91
4	$∆x_{1}$	$∆t_{4}$	30.079	2.16	12	$∆x_{3}$	$∆t_{4}$	29.701	0.88
5	$∆x_{2}$	$∆t_{1}$	30.455	3.44	13	$∆x_{4}$	$∆t_{1}$	29.939	1.69
6	$∆x_{2}$	$∆t_{2}$	30.016	1.95	14	$∆x_{4}$	$∆t_{2}$	29.616	0.59
7	$∆x_{2}$	$∆t_{3}$	29.843	1.36	15	$∆x_{4}$	$∆t_{3}$	29.448	0.02
8	$∆x_{2}$	$∆t_{4}$	29.834	1.33	16	$∆x_{4}$	$∆t_{4}$	29.442	0

**Table 8:** Grid and time-step independence test for the CFD simulation model of the disc-on-disc friction pair.

Mesh	∆x	∆t	$T_{out(i,j)}/{}^{\circ}C$	$\varepsilon_{p}$	Mesh	∆x	∆t	$T_{out(i,j)}/{}^{\circ}C$	$\varepsilon_{p}$
1	$∆x_{1}$	$∆t_{1}$	124.062	1.86	9	$∆x_{3}$	$∆t_{1}$	122.899	0.90
2	$∆x_{1}$	$∆t_{2}$	123.725	1.58	10	$∆x_{3}$	$∆t_{2}$	122.289	0.40
3	$∆x_{1}$	$∆t_{3}$	122.829	0.85	11	$∆x_{3}$	$∆t_{3}$	122.261	0.38
4	$∆x_{1}$	$∆t_{4}$	122.821	0.84	12	$∆x_{3}$	$∆t_{4}$	122.254	0.37
5	$∆x_{2}$	$∆t_{1}$	123.657	1.53	13	$∆x_{4}$	$∆t_{1}$	122.573	0.64
6	$∆x_{2}$	$∆t_{2}$	122.742	0.78	14	$∆x_{4}$	$∆t_{2}$	121.952	0.13
7	$∆x_{2}$	$∆t_{3}$	122.442	0.53	15	$∆x_{4}$	$∆t_{3}$	121.805	0.01
8	$∆x_{2}$	$∆t_{4}$	122.435	0.53	16	$∆x_{4}$	$∆t_{4}$	121.798	0

## Data Availability

The original contributions presented in this study are included in the article. Further inquiries can be directed to the corresponding author.
